# Supplementary material for: High resolution time series reveals cohesive but short-lived communities in coastal plankton
Source: Nat Commun. 2018 Jan 18;9:266. doi: 10.1038/s41467-017-02571-4 (PMC5773528; doi:10.1038/s41467-017-02571-4)
Supplement: Supplementary file 1 — Supplementary Information [file 41467_2017_2571_MOESM1_ESM.docx]

**
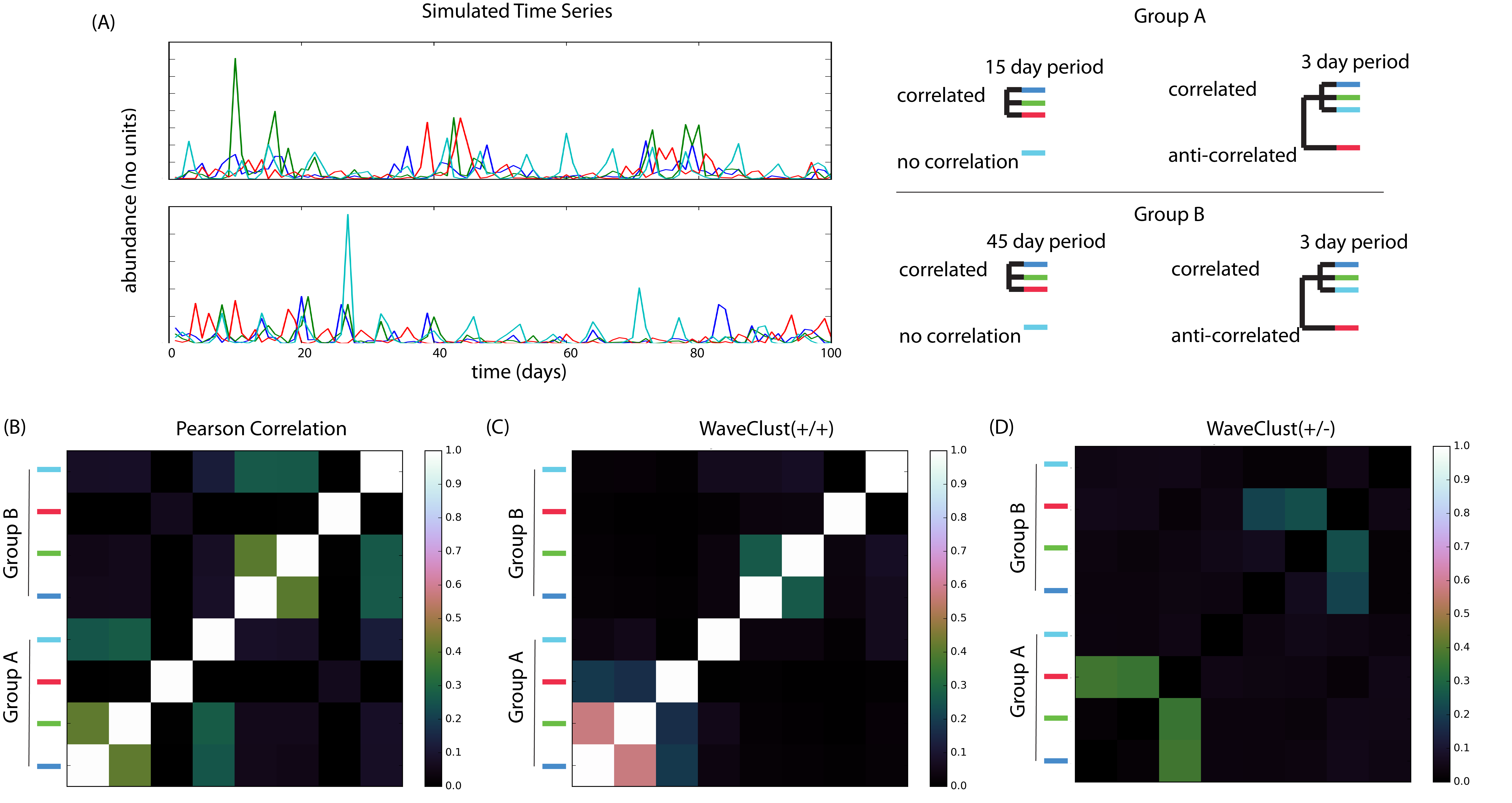
**

**Supplementary Fig. 1 |** **WaveClust performance with simulated coupled dynamics.** We simulated time series with noisy coupled dynamics, consisting of perfect coupling (correlation or anti-correlation) at one or more periods (3 day, 15 day, or 45 day) with the addition of random noise to each series (signal-to-noise ratio: 1). In panel **(A)** the blue and green series are correlated at both high and low frequency (high frequency at 3 day period, low frequency at 15 day period in “Group A” and 45 day period in “Group B”). Both blue and green are correlated with the red series at low frequency, but anti-correlated at high frequency. Thus, the pairs (blue, green) and (blue/green, red) share “emergent” periods at low frequency, but have different high frequency interactions. The teal time series is correlated at high frequency with blue and green, but shares no low frequency correlation. In panel **(B)** we calculate the pairwise Pearson correlation for all series in both Group A and Group B. While this correlation separates the two groups, it has the undesirable effect of grouping the teal series with blue and green, and it finds no relationship between blue or green and red. In panel **(C)**, WaveClust(+/+) is specifically sensitive to the (blue, green) pair, while in **(D)** WaveClust(+/-) is specifically sensitive to (blue/green, red) pairs. Similarity results reflect the average similarity calculated for each pair across 500 random trials.

**
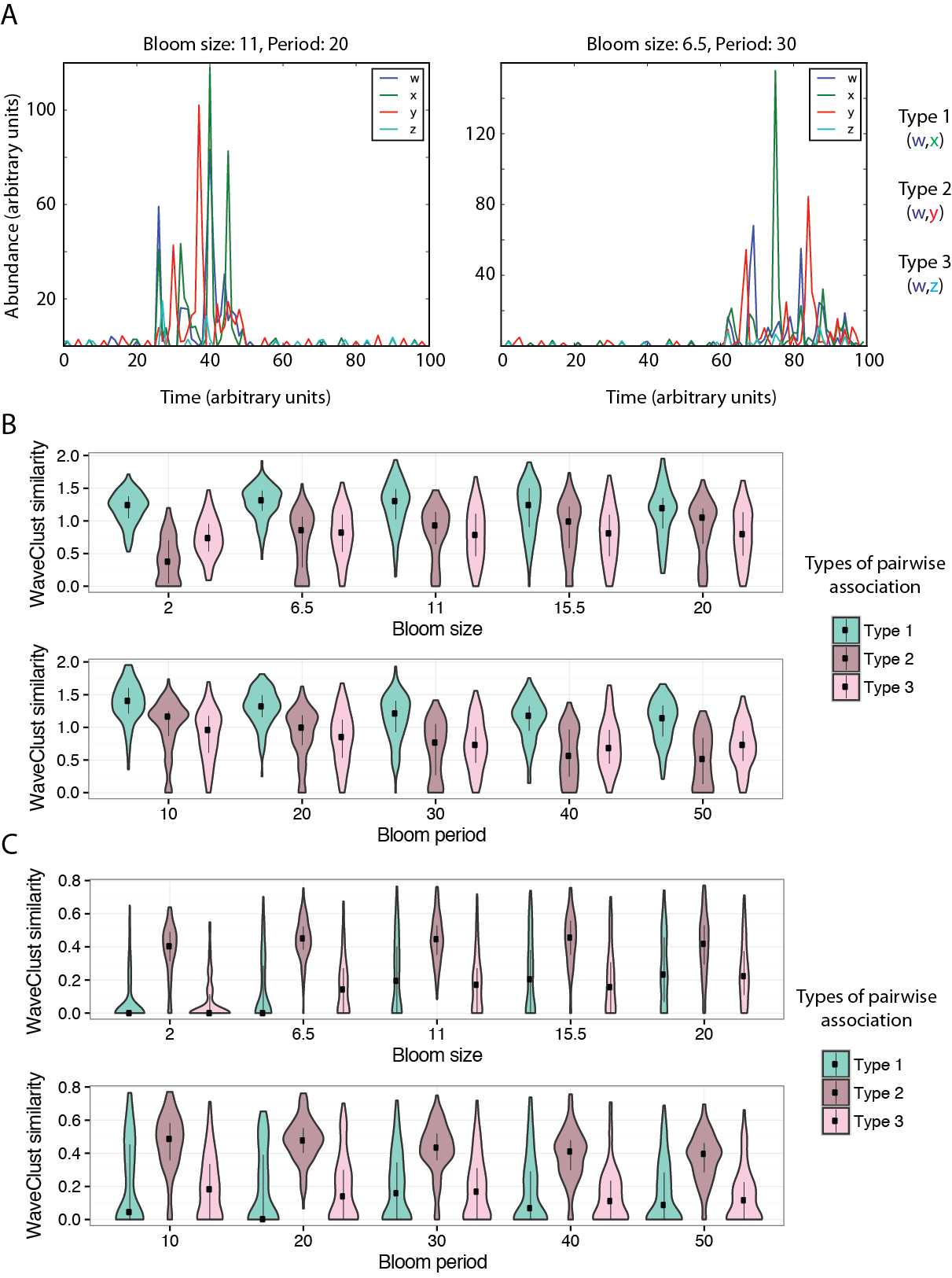
**

**Supplementary Fig. 2 | Sensitivity analysis of WaveClust similarity with simulated data.** Time series were simulated to capture patterns of blooms (occurring at low frequency and higher abundance) in the midst of basal fluctuations (occurring at high frequency and lower abundance) with the addition of random noise. Pairs of OTUs with a Type 1 association are correlated at both low and high frequency. Type 2 associations are correlated low frequency, and anti-correlated at high frequency. Type 3 associations are correlated at high frequency, and uncorrelated at low frequency. **(A)** Representative time series with different bloom size and period (left and right panels). **(B and C)** Violin plots of WaveClust similarity scores (Y axis) between pairs of simulated OTUs with Type 1, 2, or 3 associations, with varying bloom size and period (X axis). Similarity scores are calculated using positive correlation at low frequency, and either positive **(B)** or negative **(C)** correlation at low frequency. Type 1 associations are more easily detected in **(B)**, while Type 2 associations are more easily detected in **(C)**.

Supplementary Fig. 3 | Dynamics of predicted communities and metadata across the time series. (A) Communities predicted by WaveClust analysis as modular units (clusters) of interacting OTUs based on wavelet decomposition to determine correlations at different temporal frequencies followed by clustering (Materials and Methods). Shown are the results from positive correlation at low and negative correlation at high frequency. (B) Heat map displaying change in physical, biological and chemical environmental parameters over the time series. Color scale for each environmental parameter varies between maximum and minimum values, which are for each parameter: WWTMP: 20-10 ºC; ATMP: 30-10 ºC; DWD: 14-4 s, WS: 14-4 m/s; WL: 4.5-1.5 m; WH: 3-0.5 m; Press: 1020-995 hPa; TD: 1-0 incoming/outgoing; Malgae: 4-0 relative concentration; CCon: 10-4 µg/L; NH_4_: 1.4-0 µM; NO_2_-NO_3_: 20-0 µM; PO_4_: 0.7-0 µM; Silicate: 10-2 µM; Salinity: 36-33 psu. For more details see Supplementary Data 7 where values for each day are listed. Day 247, which marks hurricane Earl passage, is framed in the heat map. (C) Granger causalities linking predicted communities to each other and to environmental parameters. Nodes represent communities (colored according to panel A) and environmental parameters (grey). Legends for B and C: WTMP, water temperature; ATMP, air temperature; DWD, dominant wave period; WS, wind speed; WL, water level; WH, wave height; Press, pressure; TD, tidal direction; Malgae, macroalgae; CCon, Chlorophyll concentration; NH_4_, ammonium; NO_2_-NO_3_, nitrite and nitrates; PO_4_, phosphate.


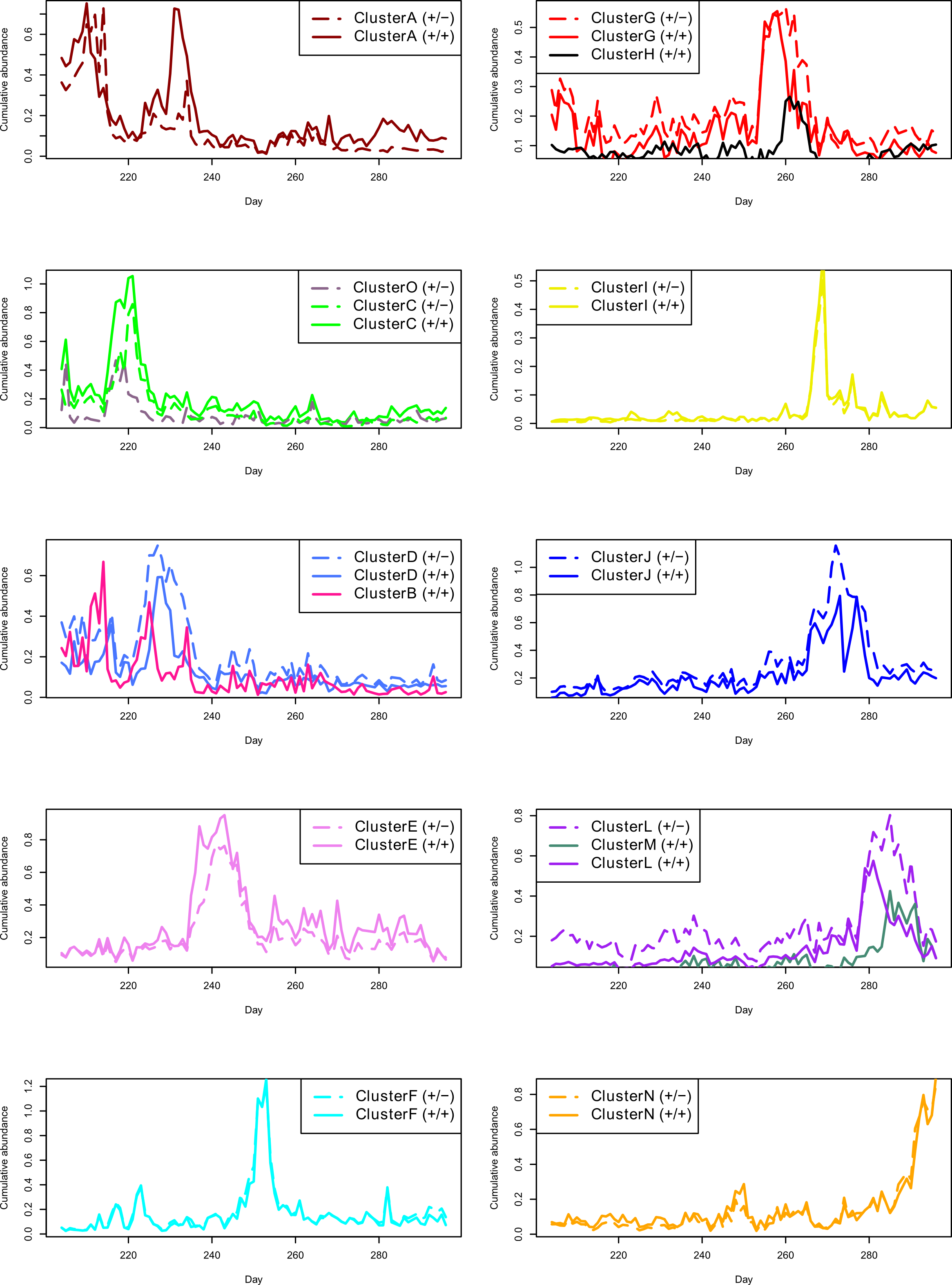


**Supplementary Fig. 4 |** **Direct comparison of communities predicted by positive correlations at low frequency with either positive (solid line) or negative (dashed line) interactions at high frequency.** Although there is generally high agreement between +/+ and +/- correlations, some exceptions emerge. For example, cluster C (+/+) approximately splits in two clusters, C and O, when considering +/- correlations. Similarly, cluster D and L in +/- correlations approximately split in two clusters (D and B, and L and M, respectively) when considering +/+ correlations. Color-coding according to communities shown in Fig. 2.


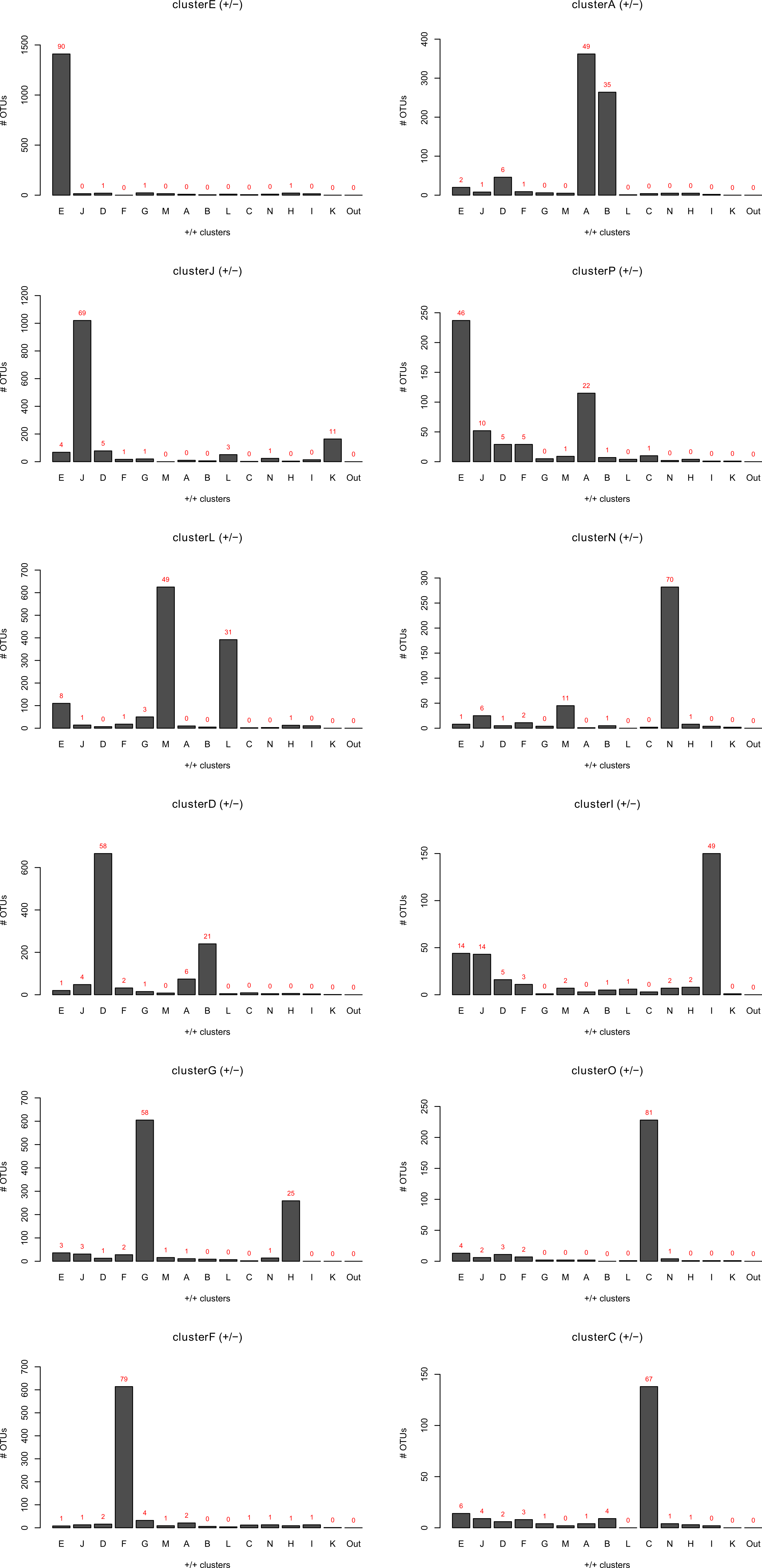


**Supplementary Figure 5 | Number of OTUs shared among clusters defined by negative and positive high frequency interactions.** For each cluster defined by negative high frequency interactions, shown is the percentage overlap in OTUs with each of the clusters defined by positive high frequency interaction ($\frac{\#intersecting OTUs}{total OTUs in cluster}$). Assigning similar clusters with more than a 65% overlap, we found that 6 out of 12 negative interaction clusters were also directly found among the positive interaction clusters, while the remaining 6 were mainly split in two of the positive clusters (both summing up more than this 65% overlap).

**
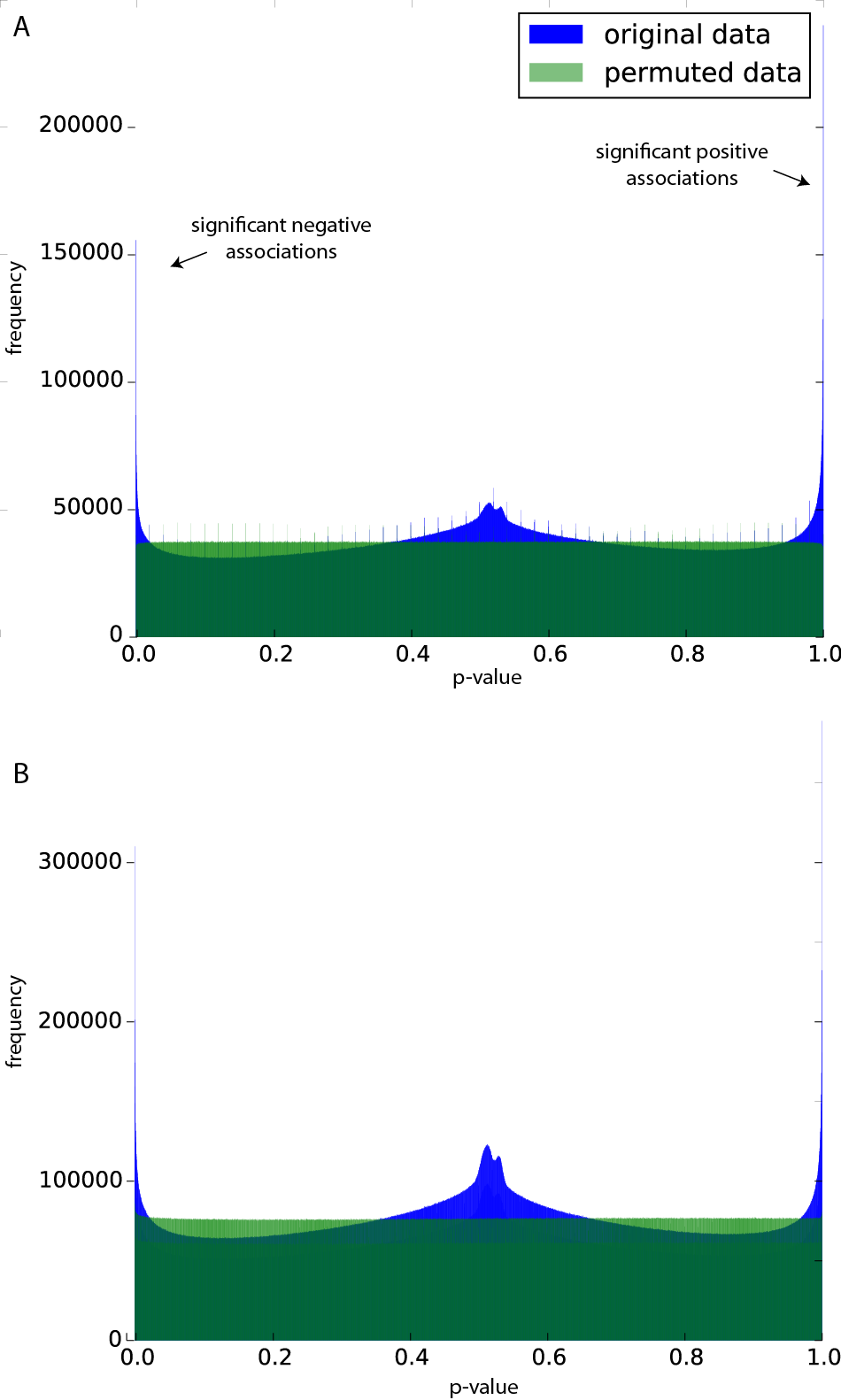
**

**Supplementary Fig. 6 | Significant positive and negative WaveClust associations identified by permutation analysis.** Permuted time series were used to calculate empirical p-values in **(A)** distributional OTU time series (original data) and **(B)** non-distributional (100% sequence identity) time series. Green histograms depict the distribution of p-values found in randomly shuffled data. Blue histograms depict the distribution of p-values in real data. Real data are enriched for significant positive (p-values close to 1) and negative associations (p-values close to 0) at a similar frequency in **(A)** and **(B)**.

**
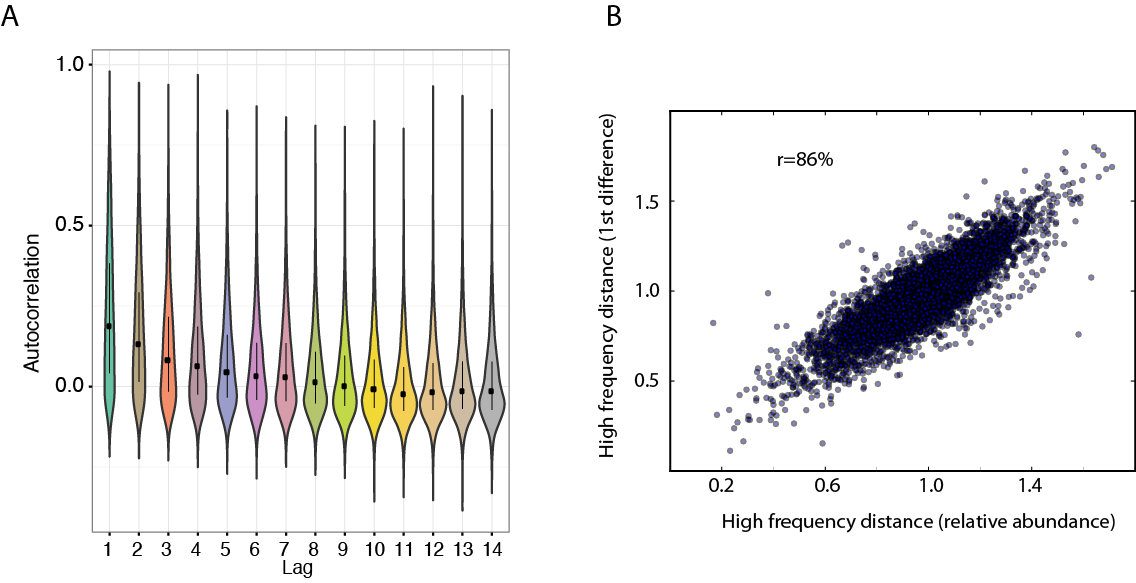
**

**Supplementary Fig. 7 | Autocorrelation has little effect on WaveClust similarity.** **(A)** Autocorrelation levels (Y axis) were calculated for each time series at a lag of 1 to 14 days (X axis). Violin plots depict the distribution of autocorrelation values at each time lag (dots represent median; bars represent interquartile range). Autocorrelation at a 1-day lag was removed by calculating the 1st difference for each time series. **(B)** High-frequency similarity scores calculated from the original data (X axis) are highly correlated (86% Pearson correlation) with high-frequency similarity scores calculated from 1st different data (Y axis; each dot represents one OTU time series).

**
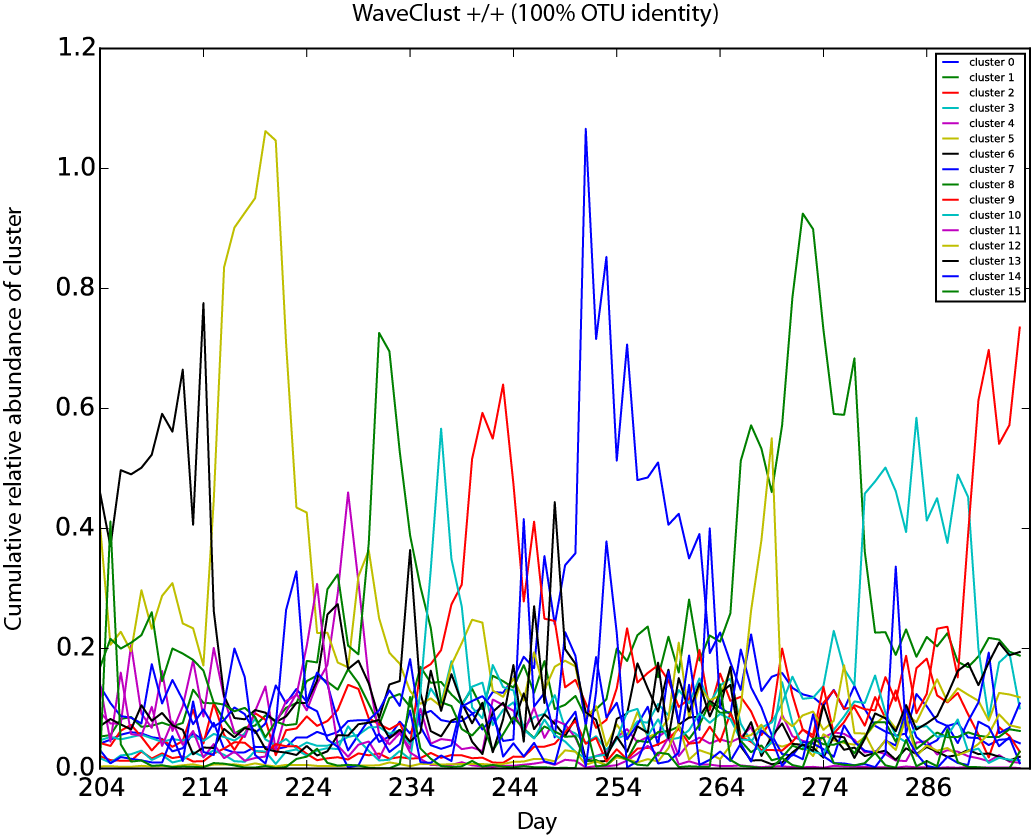
**

**Supplementary Fig. 8 | WaveClust clustering of non-distributional OTUs collapsed at 100% sequence identity.** OTUs were defined at 100% sequence identity to create an alternative dataset to distributional OTUs. Plot depicts cumulative abundance time series of non-distributional OTU clusters found by WaveClust. WaveClust scores were generated using positive correlations at high- and low-frequency (similar results were obtained with negative correlations at high-frequency; data not shown). Permutation analysis was used to keep only those scores passing a false discovery rate of 10%. The number of clusters, timing of cluster peaks, and sharp transitions are similar to results found with distributional OTUs.


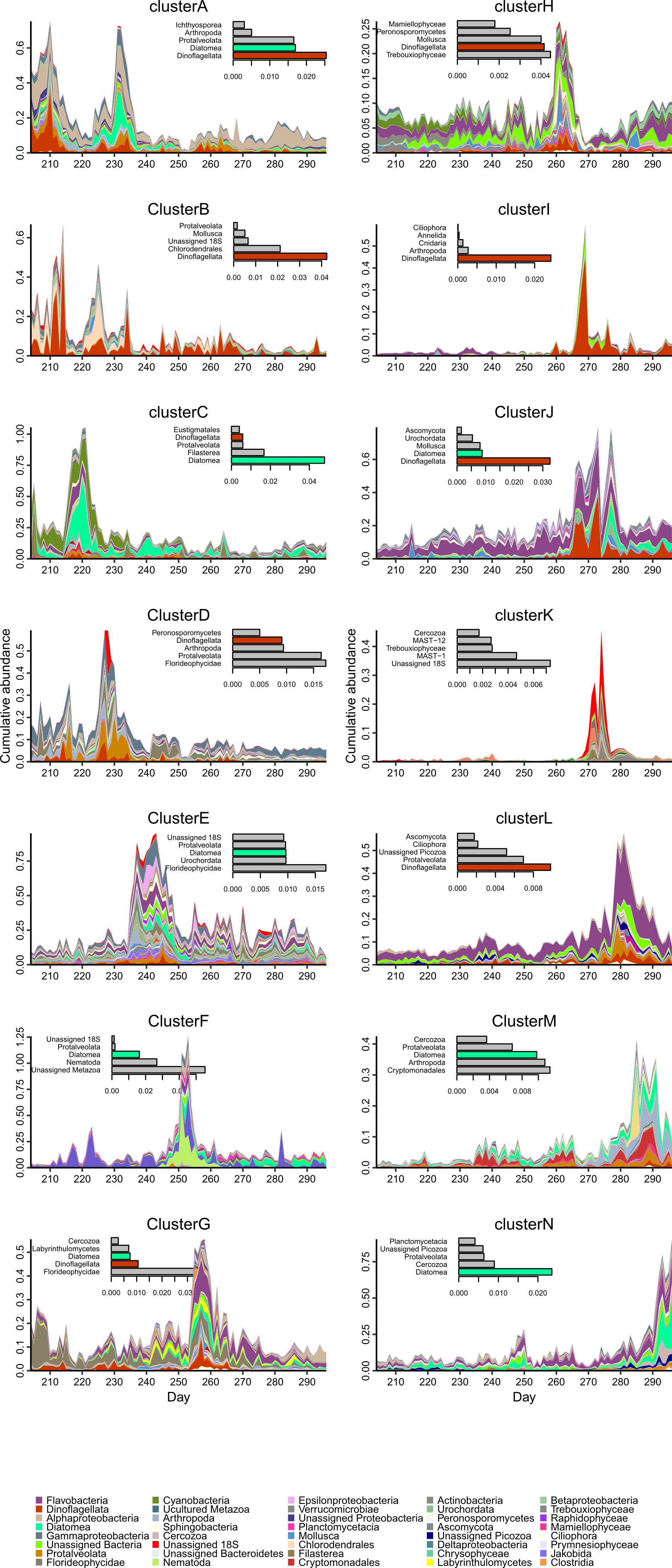


**Supplementary Fig. 9 |** **Taxonomic groups with highest relative frequencies in the most abundant communities.** Inset bargraphs show the average relative abundance of the 5 most prevalent eukaryotic groups highlighting alternating dominance of dinoflagellates (orange-red) and diatoms (spring-green) as primary producers. Legends for class-level taxa are ordered from most to least abundant for the 40 most overall abundant classes (the remaining low abundant classes are omitted in the legend).


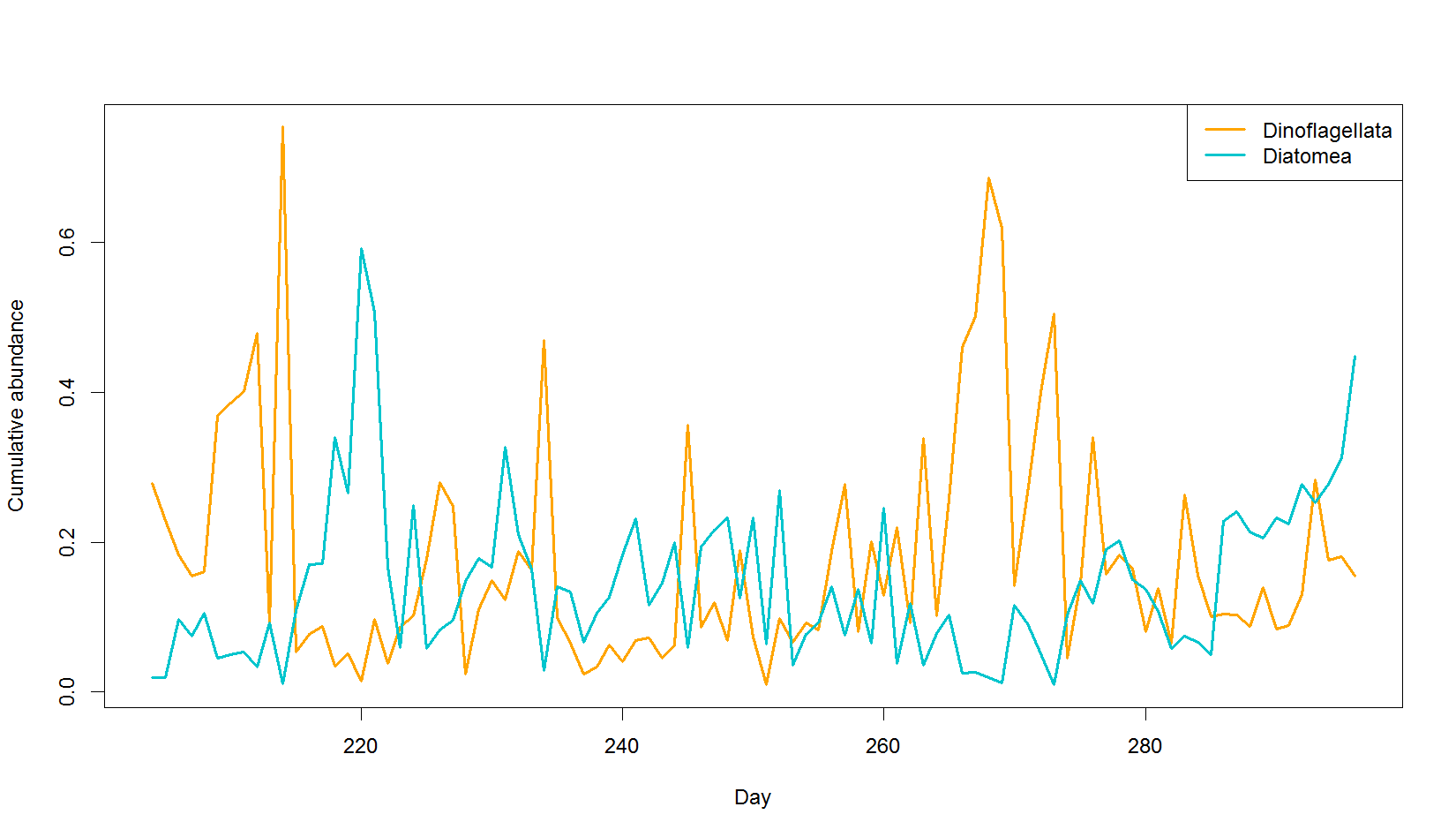


**Supplementary Fig. 10 | Alternating dominance of diatoms and dinoflagellates in the time series.** Changes in relative abundance are shown for Diatomea and Dinoflagellata, i.e. the sum of all diatom and dinoflagellate OTUs, respectively.
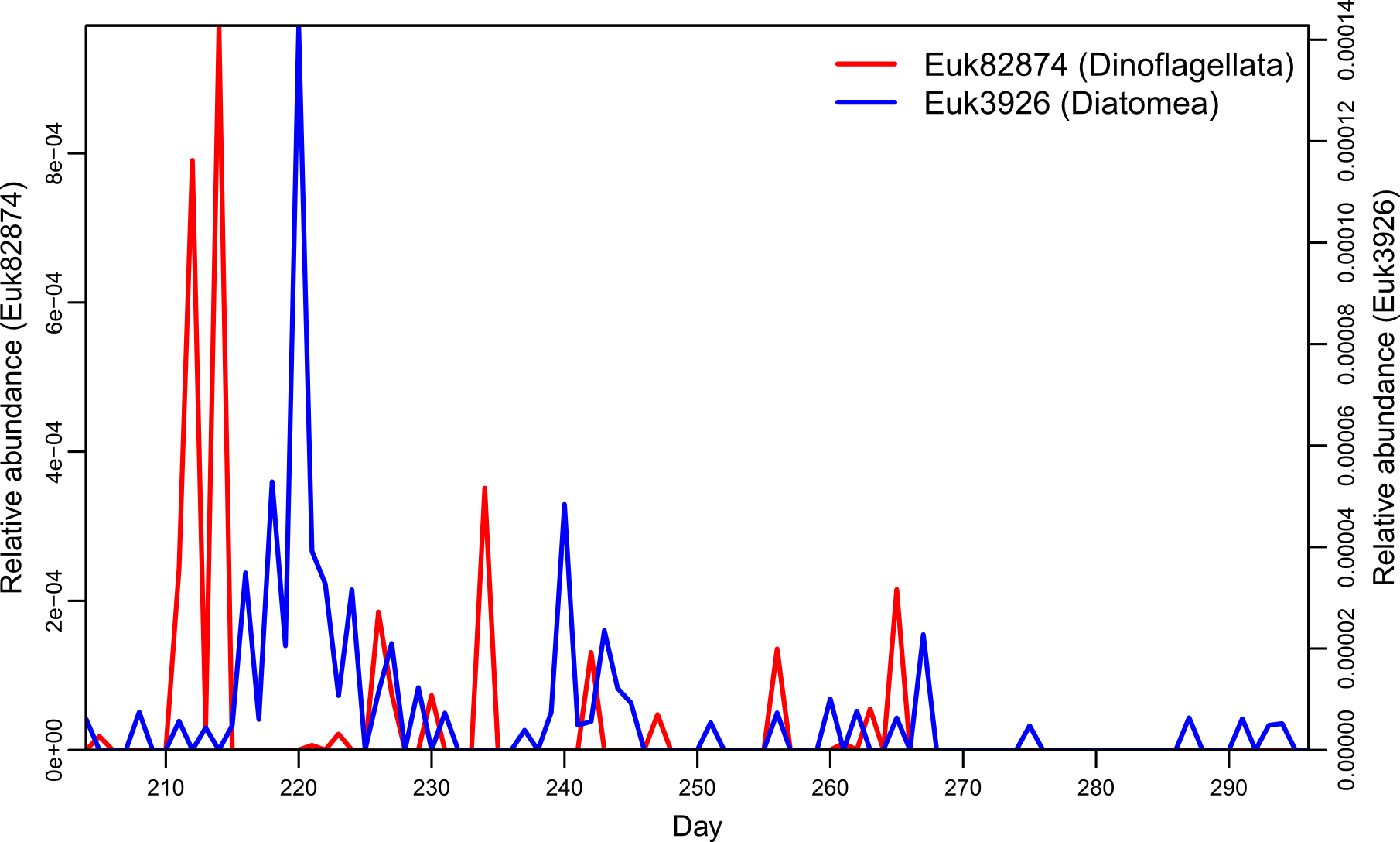


**Supplementary Fig. 11 | Example of taxonomically highly resolved alternating shifts in dominance among dinoflagellate and diatom.** Shown are changes in relative abundance among one diatom and one dinoflagellate OTU.


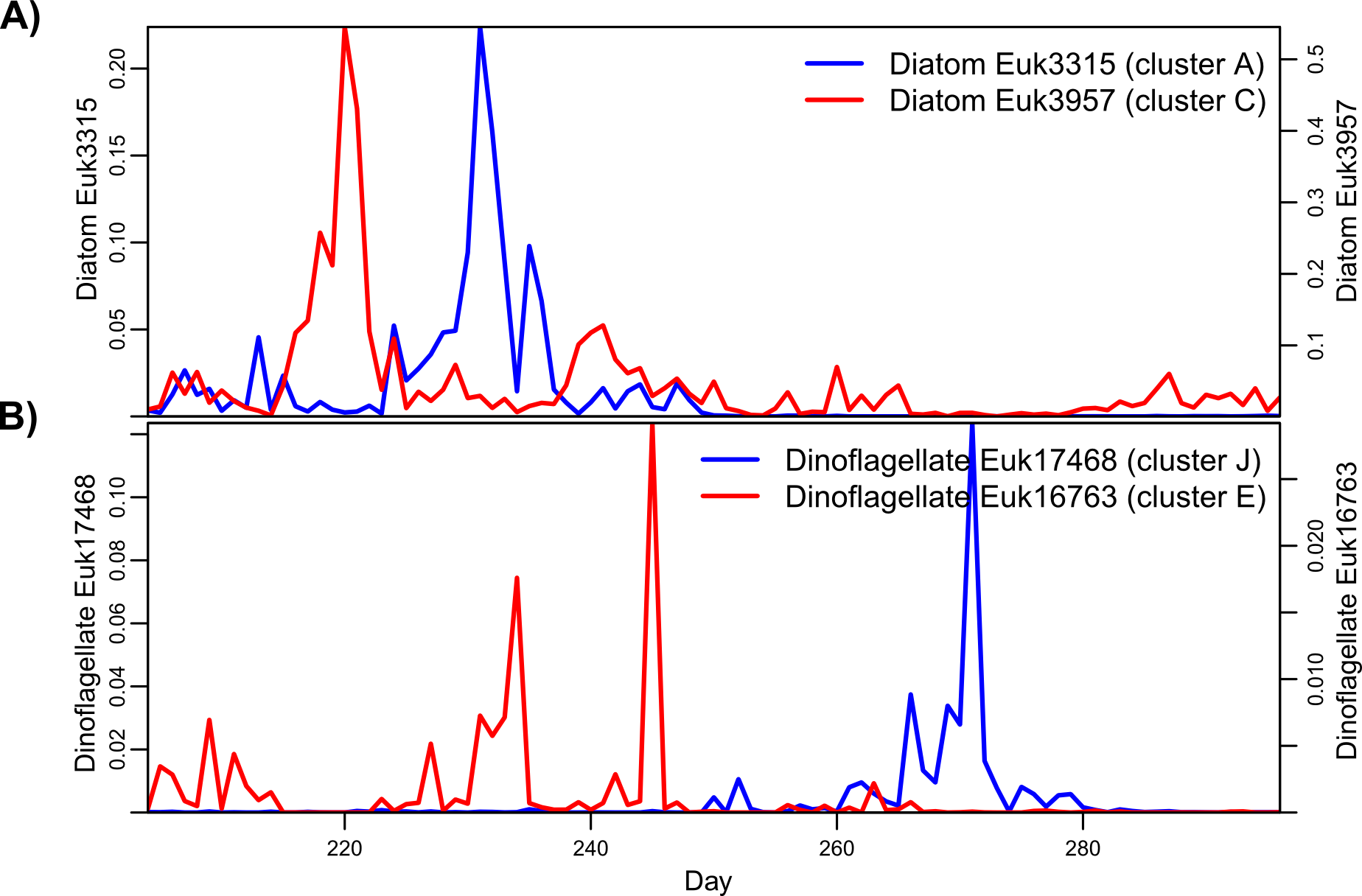


**Supplementary Fig. 12 | Different diatom and dinoflagellate OTUs peak in different communities.** Example of daily dynamics of some diatoms (A) and dinoflagellate (B) OTUs, each reaching highest relative abundance within different community clusters.

**
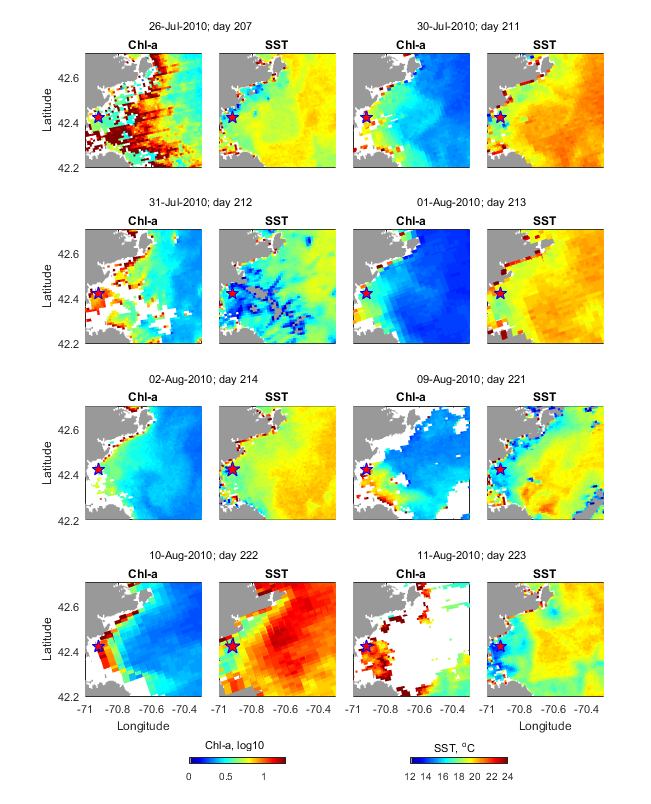
**

**
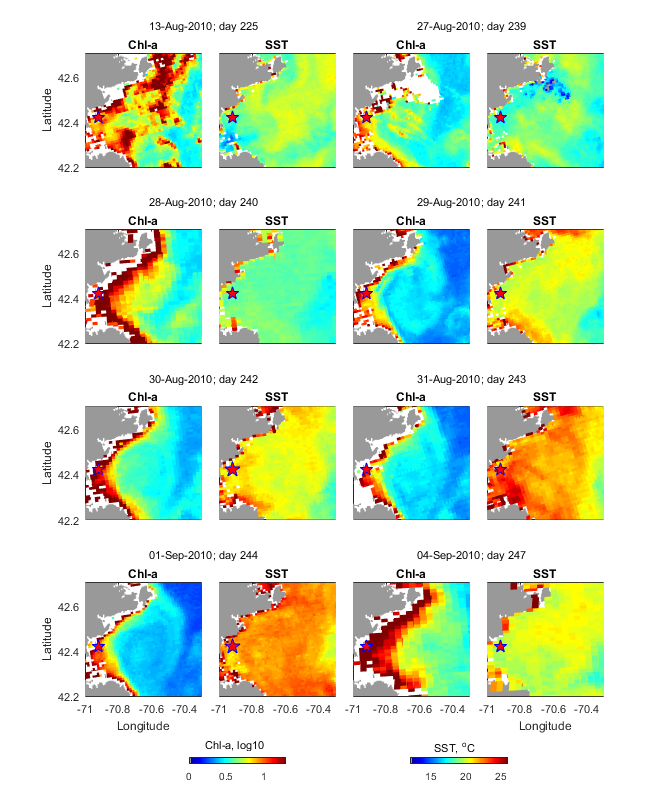
**

**
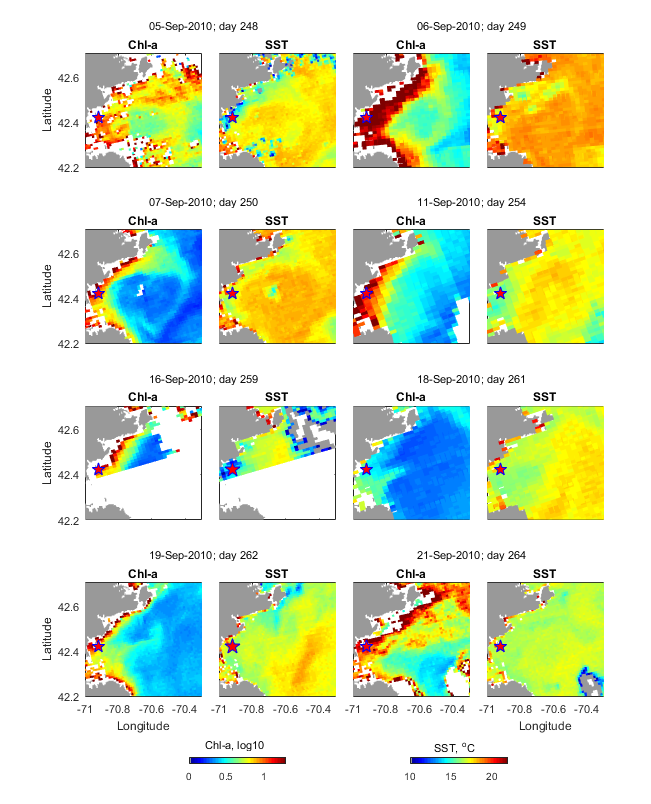
**

**
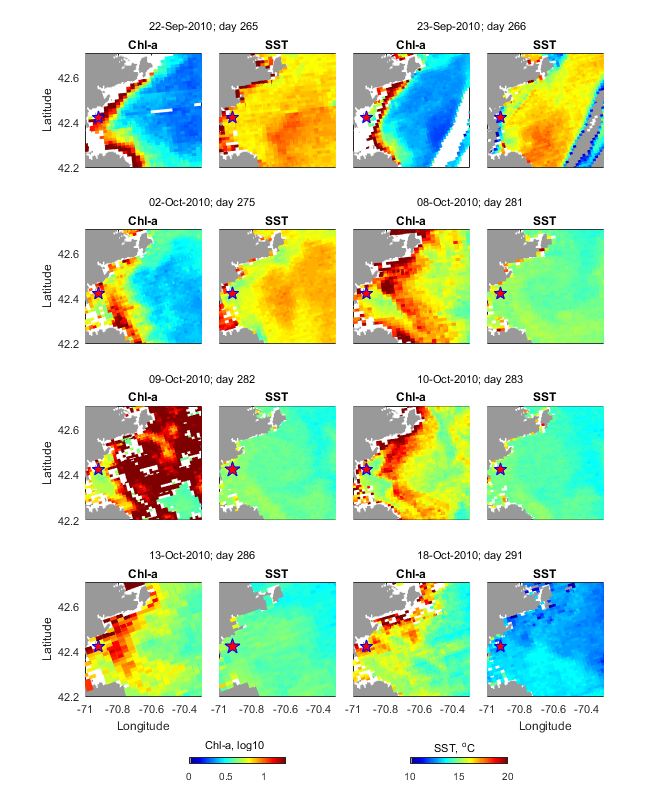
**

**
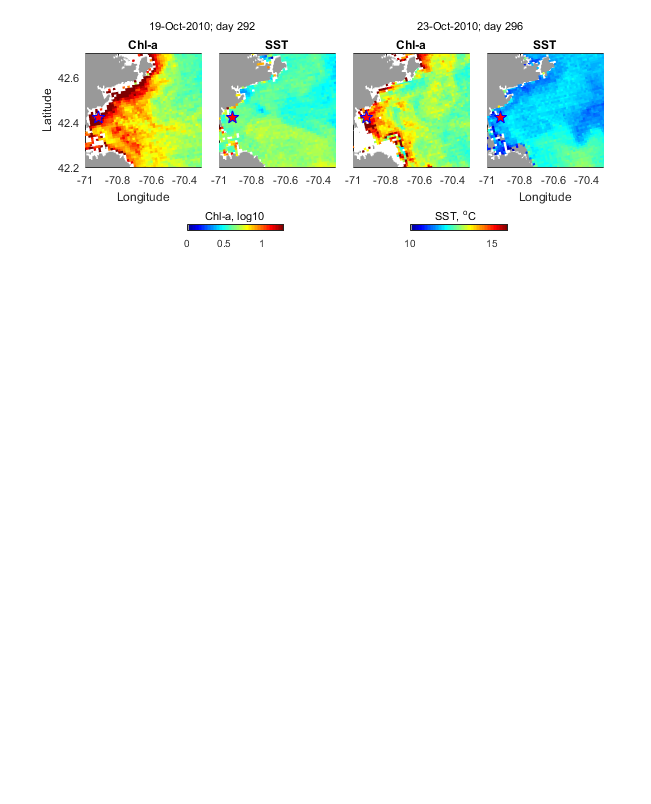
**

**Supplementary Fig. 13 | Time-series of chlorophyll *a* (Chl-a) and sea surface temperature (SST).** Data are from MODIS-A satellite imagery for the period 26 July to 23 October 2010. Only relatively cloud-free scenes are shown. Compare to Supplementary Fig. 4 for comparison of temporal dynamics of communities.

**
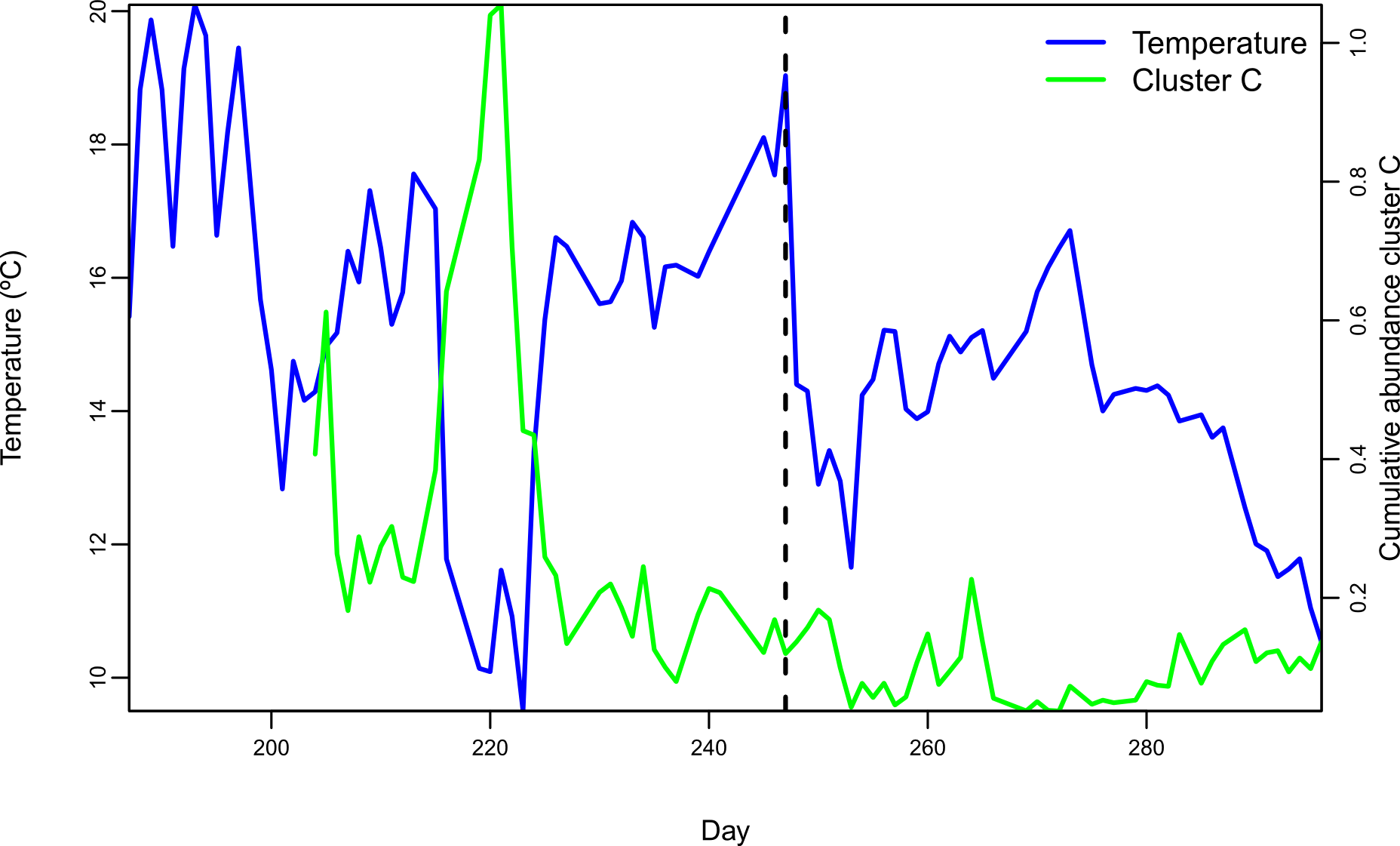
**

**Supplementary Fig. 14 | Dynamics of community cluster C and temperature variation.** Cluster C defined by positive correlations at low and high frequencies shows its highest expansion during cold water intrusion and consideration of temperature values preceding the time series sampling suggests that the slight peak at the beginning of the time series might have happened during another cold water period not captured by the biological sampling in this time series. Vertical dashed line highlights day 247, marking the passage of hurricane Earl.

**
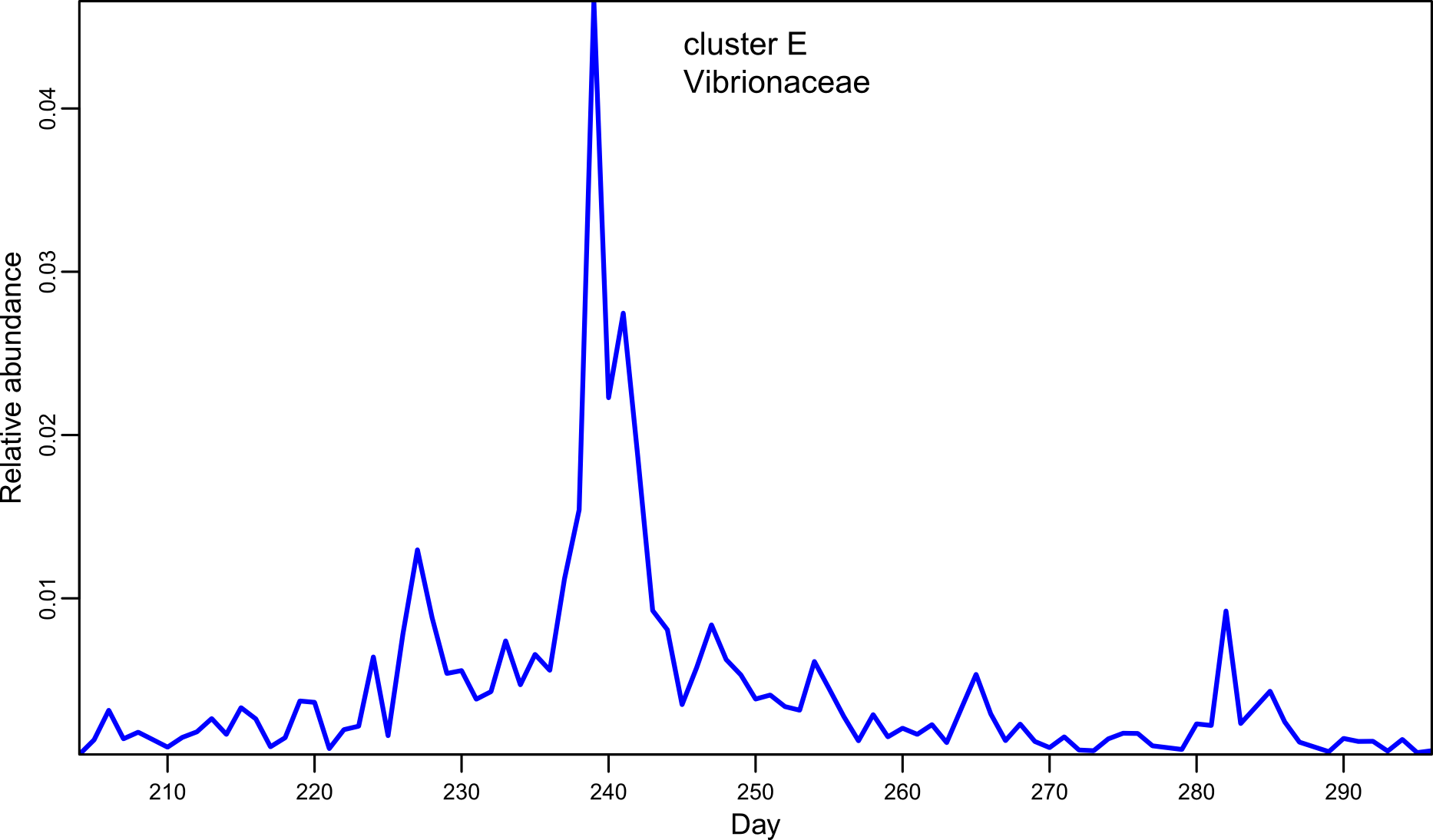
**

**Supplementary Fig. 15 | Vibrionaceae bloom in community E.** The bloom occurred during a period characterized by warm water and abundant macroalgal detritus. See Fig. 2 for details on community dynamics.


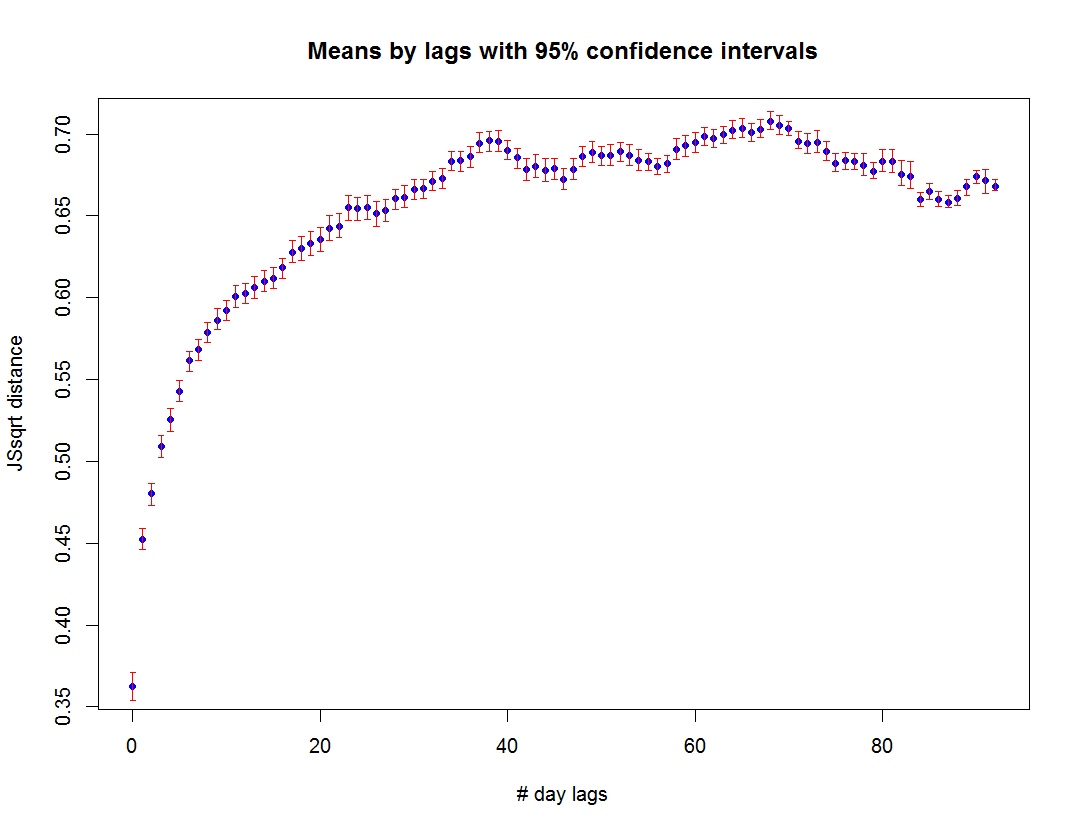


**Supplementary Fig. 16 | Jensen-Shannon square root distance between samples for different time-lags.** Replicates (lag 0) are most closely related.

**Supplementary Table 1.** Clusters estimated by WaveClust and associated numbers of OTUs.

|  | WaveClust | |
| --- | --- | --- |
| Cluster | +/+ (Fig. 2) | +/- (Fig. S3) |
| A | 622 | 737 |
| B | 560 | ND |
| C | 417 | 203 |
| D | 913 | 1133 |
| E | 1988 | 1556 |
| F | 784 | 771 |
| G | 767 | 1031 |
| H | 341 | ND |
| I | 216 | 305 |
| J | 1284 | 1478 |
| K | 171 | ND |
| L | 481 | 1260 |
| M | 743 | ND |
| N | 373 | 402 |
| O | ND | 279 |
| P | ND | 505 |

ND = cluster not detected.

**Supplementary Table 2.** Sample-specific barcode sequences.

| Barcode ID | Barcode sequence | 96-well position |
| --- | --- | --- |
| 001 | TCCGTGCGC | A1 |
| 002 | TGTTTCCCA | A2 |
| 003 | GGTAATGAA | A3 |
| 004 | GAAACTGGG | A4 |
| 005 | ACGGGCTGA | A5 |
| 006 | ATGAAGTAT | A6 |
| 007 | ACTTATTGT | A7 |
| 008 | GGCGGGAAA | A8 |
| 009 | ACACCTCGG | A9 |
| 010 | CTCATTGGG | A10 |
| 011 | GCTGCCGCG | A11 |
| 012 | CGATGGTGT | A12 |
| 013 | TCAAAGCTG | B1 |
| 014 | CAGCGGCAT | B2 |
| 015 | CCGACAAAT | B3 |
| 016 | TAAGGGAGA | B4 |
| 017 | TTGTGGCGC | B5 |
| 018 | AGGTCGGTC | B6 |
| 019 | AATGTCAAG | B7 |
| 020 | GTTCGCAGG | B8 |
| 021 | TATCAATCT | B9 |
| 022 | GTCTAACGC | B10 |
| 023 | TTACTATAC | B11 |
| 024 | TGCACCCGT | B12 |
| 025 | TGGGACCTC | C1 |
| 026 | GAGTTTGAT | C2 |
| 027 | AACAGTATT | C3 |
| 028 | ATCGCACCA | C4 |
| 029 | CTAGAATCT | C5 |
| 030 | CGCCAAGGG | C6 |
| 031 | AGTATGCAG | C7 |
| 032 | CCTTTGATA | C8 |
| 033 | TTTAACTGA | C9 |
| 034 | CTTGCTTGG | C10 |
| 035 | TCGGCTCGG | C11 |
| 036 | CAAGCCTGC | C12 |
| 037 | ATAGGTGGA | D1 |
| 038 | CAACTTCAT | D2 |
| 039 | GTAGTCGAG | D3 |
| 040 | TCCCGATGA | D4 |
| 041 | GGGCGAAAT | D5 |
| 042 | GCGTAGGAT | D6 |
| 043 | GGCCTCGCC | D7 |
| 044 | GGTGTACCA | D8 |
| 045 | CCCAGGCAG | D9 |
| 046 | GTCACGGGA | D10 |
| 047 | AATACAGGT | D11 |
| 048 | TATTCTGTA | D12 |
| 049 | CGTCCCACC | E1 |
| 050 | CTGTTAGTC | E2 |
| 051 | CACTCACTA | E3 |
| 052 | ACCTCCCAT | E4 |
| 053 | GAGCACAGG | E5 |
| 054 | CGGAGTGCT | E6 |
| 055 | GCAAGATAC | E7 |
| 056 | CGAATATTC | E8 |
| 057 | AAGGAACGT | E9 |
| 058 | GATTGAAGT | E10 |
| 059 | TGATAATAT | E11 |
| 060 | CCACGCAAG | E12 |
| 061 | TACGATACT | F1 |
| 062 | AGGCTTCAT | F2 |
| 063 | GTGCTGATC | F3 |
| 064 | ACCATACTA | F4 |
| 065 | AAATTGGAC | F5 |
| 066 | TAGAGCCAA | F6 |
| 067 | TTATCCTTG | F7 |
| 068 | TTCCAGATG | F8 |
| 069 | CACAACGAA | F9 |
| 070 | AACCCGTTG | F10 |
| 071 | CTACCGATG | F11 |
| 072 | GTGGATAGC | F12 |
| 073 | GCCTGTTCC | G1 |
| 074 | AGCTGACGG | G2 |
| 075 | AGAGAGGCT | G3 |
| 076 | GCAATGGAT | G4 |
| 077 | TGACTTAGC | G5 |
| 078 | AAACAAGAT | G6 |
| 079 | CTTCAGCTG | G7 |
| 080 | GGAGGCTGT | G8 |
| 081 | ACAAACTAC | G9 |
| 082 | GACATCATA | G10 |
| 083 | AGTCACCCG | G11 |
| 084 | TCTAGTCGT | G12 |
| 085 | CCGCACCGA | H1 |
| 086 | ATGCCAGCA | H2 |
| 087 | TCGAACACA | H3 |
| 088 | CGACATTCA | H4 |
| 089 | CATCGCTAG | H5 |
| 090 | AAATCATTA | H6 |
| 091 | TCTGTATGT | H7 |
| 092 | ACTAAGATA | H8 |
| 093 | CCCGTTTCA | H9 |
| 094 | GTACGTTGC | H10 |
| 095 | AGTAGATGA | H11 |
| 096 | TCATTAAGG | H12 |

**Supplementary Table 3. Sequencing platform used for each sample.**

| Sample | rRNA gene sequenced | Sequencing platform |
| --- | --- | --- |
| 10N.204.37 | 16S | Illumina MiSeq |
| 10N.204.38 | 16S | Illumina MiSeq |
| 10N.204.39 | 16S | Illumina MiSeq |
| 10N.205.37 | 16S | Illumina MiSeq |
| 10N.205.38 | 16S | Illumina MiSeq |
| 10N.205.39 | 16S | Illumina MiSeq |
| 10N.206.37 | 16S | Illumina MiSeq |
| 10N.206.38 | 16S | Illumina MiSeq |
| 10N.206.39 | 16S | Illumina MiSeq |
| 10N.207.37 | 16S | Illumina MiSeq |
| 10N.207.38 | 16S | Illumina MiSeq |
| 10N.207.39 | 16S | Illumina MiSeq |
| 10N.208.37 | 16S | Illumina MiSeq |
| 10N.208.38 | 16S | Illumina MiSeq |
| 10N.208.39 | 16S | Illumina MiSeq |
| 10N.209.37 | 16S | Illumina MiSeq |
| 10N.209.38 | 16S | Illumina MiSeq |
| 10N.209.39 | 16S | Illumina MiSeq |
| 10N.210.37 | 16S | Illumina MiSeq |
| 10N.210.38 | 16S | Illumina MiSeq |
| 10N.210.39 | 16S | Illumina MiSeq |
| 10N.211.37 | 16S | Illumina MiSeq |
| 10N.211.38 | 16S | Illumina MiSeq |
| 10N.211.39 | 16S | Illumina MiSeq |
| 10N.212.37 | 16S | Illumina MiSeq |
| 10N.212.38 | 16S | Illumina MiSeq |
| 10N.212.39 | 16S | Illumina MiSeq |
| 10N.213.37 | 16S | Illumina MiSeq |
| 10N.213.38 | 16S | Illumina MiSeq |
| 10N.213.39 | 16S | Illumina MiSeq |
| 10N.214.37 | 16S | Illumina MiSeq |
| 10N.214.38 | 16S | Illumina MiSeq |
| 10N.214.39 | 16S | Illumina MiSeq |
| 10N.215.37 | 16S | Illumina MiSeq |
| 10N.215.38 | 16S | Illumina MiSeq |
| 10N.215.39 | 16S | Illumina MiSeq |
| 10N.216.37 | 16S | Illumina MiSeq |
| 10N.216.38 | 16S | Illumina MiSeq |
| 10N.216.39 | 16S | Illumina MiSeq |
| 10N.217.37 | 16S | Illumina MiSeq |
| 10N.217.38 | 16S | Illumina MiSeq |
| 10N.217.39 | 16S | Illumina MiSeq |
| 10N.218.37 | 16S | Illumina MiSeq |
| 10N.218.38 | 16S | Illumina MiSeq |
| 10N.218.39 | 16S | Illumina MiSeq |
| 10N.219.37 | 16S | Illumina MiSeq |
| 10N.219.38 | 16S | Illumina MiSeq |
| 10N.219.39 | 16S | Illumina MiSeq |
| 10N.220.37 | 16S | Illumina MiSeq |
| 10N.220.38 | 16S | Illumina MiSeq |
| 10N.220.39 | 16S | Illumina MiSeq |
| 10N.221.37 | 16S | Illumina MiSeq |
| 10N.221.38 | 16S | Illumina MiSeq |
| 10N.221.39 | 16S | Illumina MiSeq |
| 10N.222.37 | 16S | Illumina MiSeq |
| 10N.222.38 | 16S | Illumina MiSeq |
| 10N.222.39 | 16S | Illumina MiSeq |
| 10N.223.37 | 16S | Illumina MiSeq |
| 10N.223.38 | 16S | Illumina MiSeq |
| 10N.223.39 | 16S | Illumina MiSeq |
| 10N.224.37 | 16S | Illumina MiSeq |
| 10N.224.38 | 16S | Illumina MiSeq |
| 10N.224.39 | 16S | Illumina MiSeq |
| 10N.225.37 | 16S | Illumina MiSeq |
| 10N.225.38 | 16S | Illumina MiSeq |
| 10N.225.39 | 16S | Illumina MiSeq |
| 10N.226.37 | 16S | Illumina MiSeq |
| 10N.226.38 | 16S | Illumina MiSeq |
| 10N.226.39 | 16S | Illumina MiSeq |
| 10N.227.37 | 16S | Illumina MiSeq |
| 10N.227.38 | 16S | Illumina MiSeq |
| 10N.227.39 | 16S | Illumina MiSeq |
| 10N.228.37 | 16S | Illumina MiSeq |
| 10N.228.38 | 16S | Illumina MiSeq |
| 10N.228.39 | 16S | Illumina MiSeq |
| 10N.229.37 | 16S | Illumina MiSeq |
| 10N.229.38 | 16S | Illumina MiSeq |
| 10N.229.39 | 16S | Illumina MiSeq |
| 10N.230.37 | 16S | Illumina MiSeq |
| 10N.230.38 | 16S | Illumina MiSeq |
| 10N.230.39 | 16S | Illumina MiSeq |
| 10N.231.37 | 16S | Illumina MiSeq |
| 10N.231.38 | 16S | Illumina MiSeq |
| 10N.231.39 | 16S | Illumina MiSeq |
| 10N.232.37 | 16S | Illumina MiSeq |
| 10N.232.38 | 16S | Illumina MiSeq |
| 10N.232.39 | 16S | Illumina MiSeq |
| 10N.233.37 | 16S | Illumina MiSeq |
| 10N.233.38 | 16S | Illumina MiSeq |
| 10N.233.39 | 16S | Illumina MiSeq |
| 10N.234.37 | 16S | Illumina MiSeq |
| 10N.234.38 | 16S | Illumina MiSeq |
| 10N.234.39 | 16S | Illumina MiSeq |
| 10N.235.37 | 16S | Illumina MiSeq |
| 10N.235.38 | 16S | Illumina MiSeq |
| 10N.235.39 | 16S | Illumina HiSeq 1000 |
| 10N.236.37 | 16S | Illumina HiSeq 1000 |
| 10N.236.38 | 16S | Illumina HiSeq 1000 |
| 10N.236.39 | 16S | Illumina HiSeq 1000 |
| 10N.237.37 | 16S | Illumina HiSeq 1000 |
| 10N.237.38 | 16S | Illumina HiSeq 1000 |
| 10N.237.39 | 16S | Illumina HiSeq 1000 |
| 10N.238.37 | 16S | Illumina HiSeq 1000 |
| 10N.238.38 | 16S | Illumina HiSeq 1000 |
| 10N.238.39 | 16S | Illumina HiSeq 1000 |
| 10N.239.37 | 16S | Illumina HiSeq 1000 |
| 10N.239.38 | 16S | Illumina HiSeq 1000 |
| 10N.239.39 | 16S | Illumina HiSeq 1000 |
| 10N.240.37 | 16S | Illumina HiSeq 1000 |
| 10N.240.38 | 16S | Illumina HiSeq 1000 |
| 10N.240.39 | 16S | Illumina HiSeq 1000 |
| 10N.241.37 | 16S | Illumina HiSeq 1000 |
| 10N.241.38 | 16S | Illumina HiSeq 1000 |
| 10N.241.39 | 16S | Illumina HiSeq 1000 |
| 10N.242.37 | 16S | Illumina HiSeq 1000 |
| 10N.242.38 | 16S | Illumina HiSeq 1000 |
| 10N.242.39 | 16S | Illumina HiSeq 1000 |
| 10N.243.37 | 16S | Illumina HiSeq 1000 |
| 10N.243.38 | 16S | Illumina HiSeq 1000 |
| 10N.243.39 | 16S | Illumina HiSeq 1000 |
| 10N.244.37 | 16S | Illumina HiSeq 1000 |
| 10N.244.38 | 16S | Illumina HiSeq 1000 |
| 10N.244.39 | 16S | Illumina HiSeq 1000 |
| 10N.245.37 | 16S | Illumina HiSeq 1000 |
| 10N.245.38 | 16S | Illumina HiSeq 1000 |
| 10N.245.39 | 16S | Illumina HiSeq 1000 |
| 10N.246.37 | 16S | Illumina HiSeq 1000 |
| 10N.246.38 | 16S | Illumina HiSeq 1000 |
| 10N.246.39 | 16S | Illumina HiSeq 1000 |
| 10N.247.37 | 16S | Illumina HiSeq 1000 |
| 10N.247.38 | 16S | Illumina HiSeq 1000 |
| 10N.247.39 | 16S | Illumina HiSeq 1000 |
| 10N.248.37 | 16S | Illumina HiSeq 1000 |
| 10N.248.38 | 16S | Illumina HiSeq 1000 |
| 10N.248.39 | 16S | Illumina HiSeq 1000 |
| 10N.249.37 | 16S | Illumina HiSeq 1000 |
| 10N.249.38 | 16S | Illumina HiSeq 1000 |
| 10N.249.39 | 16S | Illumina HiSeq 1000 |
| 10N.250.37 | 16S | Illumina HiSeq 1000 |
| 10N.250.38 | 16S | Illumina HiSeq 1000 |
| 10N.250.39 | 16S | Illumina HiSeq 1000 |
| 10N.251.37 | 16S | Illumina HiSeq 1000 |
| 10N.251.38 | 16S | Illumina HiSeq 1000 |
| 10N.251.39 | 16S | Illumina HiSeq 1000 |
| 10N.252.37 | 16S | Illumina HiSeq 1000 |
| 10N.252.38 | 16S | Illumina HiSeq 1000 |
| 10N.252.39 | 16S | Illumina HiSeq 1000 |
| 10N.253.37 | 16S | Illumina HiSeq 1000 |
| 10N.253.38 | 16S | Illumina HiSeq 1000 |
| 10N.253.39 | 16S | Illumina HiSeq 1000 |
| 10N.254.37 | 16S | Illumina HiSeq 1000 |
| 10N.254.38 | 16S | Illumina HiSeq 1000 |
| 10N.254.39 | 16S | Illumina HiSeq 1000 |
| 10N.255.37 | 16S | Illumina HiSeq 1000 |
| 10N.255.38 | 16S | Illumina HiSeq 1000 |
| 10N.255.39 | 16S | Illumina HiSeq 1000 |
| 10N.256.37 | 16S | Illumina HiSeq 1000 |
| 10N.256.38 | 16S | Illumina HiSeq 1000 |
| 10N.256.39 | 16S | Illumina HiSeq 1000 |
| 10N.257.37 | 16S | Illumina HiSeq 1000 |
| 10N.257.38 | 16S | Illumina HiSeq 1000 |
| 10N.257.39 | 16S | Illumina HiSeq 1000 |
| 10N.258.37 | 16S | Illumina HiSeq 1000 |
| 10N.258.38 | 16S | Illumina HiSeq 1000 |
| 10N.258.39 | 16S | Illumina HiSeq 1000 |
| 10N.259.37 | 16S | Illumina HiSeq 1000 |
| 10N.259.38 | 16S | Illumina HiSeq 1000 |
| 10N.259.39 | 16S | Illumina HiSeq 1000 |
| 10N.260.37 | 16S | Illumina HiSeq 1000 |
| 10N.260.38 | 16S | Illumina HiSeq 1000 |
| 10N.260.39 | 16S | Illumina HiSeq 1000 |
| 10N.261.37 | 16S | Illumina HiSeq 1000 |
| 10N.261.38 | 16S | Illumina HiSeq 1000 |
| 10N.261.39 | 16S | Illumina HiSeq 1000 |
| 10N.262.37 | 16S | Illumina HiSeq 1000 |
| 10N.262.38 | 16S | Illumina HiSeq 1000 |
| 10N.262.39 | 16S | Illumina HiSeq 1000 |
| 10N.263.37 | 16S | Illumina HiSeq 1000 |
| 10N.263.38 | 16S | Illumina HiSeq 1000 |
| 10N.263.39 | 16S | Illumina HiSeq 1000 |
| 10N.264.37 | 16S | Illumina HiSeq 1000 |
| 10N.264.38 | 16S | Illumina HiSeq 1000 |
| 10N.264.39 | 16S | Illumina HiSeq 1000 |
| 10N.265.37 | 16S | Illumina HiSeq 1000 |
| 10N.265.38 | 16S | Illumina HiSeq 1000 |
| 10N.265.39 | 16S | Illumina HiSeq 1000 |
| 10N.266.37 | 16S | Illumina HiSeq 1000 |
| 10N.266.38 | 16S | Illumina HiSeq 1000 |
| 10N.266.39 | 16S | Illumina HiSeq 1000 |
| 10N.267.37 | 16S | Illumina HiSeq 1000 |
| 10N.267.38 | 16S | Illumina HiSeq 1000 |
| 10N.267.39 | 16S | Illumina HiSeq 1000 |
| 10N.268.37 | 16S | Illumina HiSeq 1000 |
| 10N.268.38 | 16S | Illumina HiSeq 1000 |
| 10N.268.39 | 16S | Illumina HiSeq 1000 |
| 10N.269.37 | 16S | Illumina HiSeq 1000 |
| 10N.269.38 | 16S | Illumina HiSeq 1000 |
| 10N.269.39 | 16S | Illumina HiSeq 1000 |
| 10N.270.37 | 16S | Illumina HiSeq 1000 |
| 10N.270.38 | 16S | Illumina HiSeq 1000 |
| 10N.270.39 | 16S | Illumina HiSeq 1000 |
| 10N.271.37 | 16S | Illumina HiSeq 1000 |
| 10N.271.38 | 16S | Illumina HiSeq 1000 |
| 10N.271.39 | 16S | Illumina HiSeq 1000 |
| 10N.272.37 | 16S | Illumina HiSeq 1000 |
| 10N.272.38 | 16S | Illumina HiSeq 1000 |
| 10N.272.39 | 16S | Illumina HiSeq 1000 |
| 10N.273.37 | 16S | Illumina HiSeq 1000 |
| 10N.273.38 | 16S | Illumina HiSeq 1000 |
| 10N.273.39 | 16S | Illumina HiSeq 1000 |
| 10N.274.37 | 16S | Illumina HiSeq 1000 |
| 10N.274.38 | 16S | Illumina HiSeq 1000 |
| 10N.274.39 | 16S | Illumina HiSeq 1000 |
| 10N.275.37 | 16S | Illumina HiSeq 1000 |
| 10N.275.38 | 16S | Illumina HiSeq 1000 |
| 10N.275.39 | 16S | Illumina HiSeq 1000 |
| 10N.276.37 | 16S | Illumina HiSeq 1000 |
| 10N.276.38 | 16S | Illumina HiSeq 1000 |
| 10N.276.39 | 16S | Illumina HiSeq 1000 |
| 10N.277.37 | 16S | Illumina HiSeq 1000 |
| 10N.277.38 | 16S | Illumina HiSeq 1000 |
| 10N.277.39 | 16S | Illumina HiSeq 1000 |
| 10N.278.37 | 16S | Illumina HiSeq 1000 |
| 10N.278.38 | 16S | Illumina HiSeq 1000 |
| 10N.278.39 | 16S | Illumina HiSeq 1000 |
| 10N.279.37 | 16S | Illumina HiSeq 1000 |
| 10N.279.38 | 16S | Illumina HiSeq 1000 |
| 10N.279.39 | 16S | Illumina HiSeq 1000 |
| 10N.280.37 | 16S | Illumina HiSeq 1000 |
| 10N.280.38 | 16S | Illumina HiSeq 1000 |
| 10N.280.39 | 16S | Illumina HiSeq 1000 |
| 10N.281.37 | 16S | Illumina HiSeq 1000 |
| 10N.281.38 | 16S | Illumina HiSeq 1000 |
| 10N.281.39 | 16S | Illumina HiSeq 1000 |
| 10N.282.37 | 16S | Illumina HiSeq 1000 |
| 10N.282.38 | 16S | Illumina HiSeq 1000 |
| 10N.282.39 | 16S | Illumina HiSeq 1000 |
| 10N.283.37 | 16S | Illumina HiSeq 1000 |
| 10N.283.38 | 16S | Illumina HiSeq 1000 |
| 10N.283.39 | 16S | Illumina HiSeq 1000 |
| 10N.284.37 | 16S | Illumina HiSeq 1000 |
| 10N.284.38 | 16S | Illumina HiSeq 1000 |
| 10N.284.39 | 16S | Illumina HiSeq 1000 |
| 10N.285.37 | 16S | Illumina HiSeq 1000 |
| 10N.285.38 | 16S | Illumina HiSeq 1000 |
| 10N.285.39 | 16S | Illumina HiSeq 1000 |
| 10N.286.37 | 16S | Illumina HiSeq 1000 |
| 10N.286.38 | 16S | Illumina HiSeq 1000 |
| 10N.286.39 | 16S | Illumina HiSeq 1000 |
| 10N.287.37 | 16S | Illumina HiSeq 1000 |
| 10N.287.38 | 16S | Illumina HiSeq 1000 |
| 10N.287.39 | 16S | Illumina HiSeq 1000 |
| 10N.288.37 | 16S | Illumina HiSeq 1000 |
| 10N.288.38 | 16S | Illumina HiSeq 1000 |
| 10N.288.39 | 16S | Illumina HiSeq 1000 |
| 10N.289.37 | 16S | Illumina HiSeq 1000 |
| 10N.289.38 | 16S | Illumina HiSeq 1000 |
| 10N.289.39 | 16S | Illumina HiSeq 1000 |
| 10N.290.37 | 16S | Illumina HiSeq 1000 |
| 10N.290.38 | 16S | Illumina HiSeq 1000 |
| 10N.290.39 | 16S | Illumina HiSeq 1000 |
| 10N.291.37 | 16S | Illumina HiSeq 1000 |
| 10N.291.38 | 16S | Illumina HiSeq 1000 |
| 10N.291.39 | 16S | Illumina HiSeq 1000 |
| 10N.292.37 | 16S | Illumina HiSeq 1000 |
| 10N.292.38 | 16S | Illumina HiSeq 1000 |
| 10N.292.39 | 16S | Illumina HiSeq 1000 |
| 10N.293.37 | 16S | Illumina HiSeq 1000 |
| 10N.293.38 | 16S | Illumina HiSeq 1000 |
| 10N.293.39 | 16S | Illumina HiSeq 1000 |
| 10N.294.37 | 16S | Illumina HiSeq 1000 |
| 10N.294.38 | 16S | Illumina HiSeq 1000 |
| 10N.294.39 | 16S | Illumina HiSeq 1000 |
| 10N.295.37 | 16S | Illumina HiSeq 1000 |
| 10N.295.38 | 16S | Illumina HiSeq 1000 |
| 10N.295.39 | 16S | Illumina HiSeq 1000 |
| 10N.296.37 | 16S | Illumina HiSeq 1000 |
| 10N.296.38 | 16S | Illumina HiSeq 1000 |
| 10N.296.39 | 16S | Illumina HiSeq 1000 |

**Supplementary Table 4.** Inflation values used in MCL clustering and effect on OTU inclusion in clusters.

| **Inflation** | **Fraction in** | |
| --- | --- | --- |
|  | **+/+ Clusters** | **+/- Clusters** |
| 1.6 | 100% | 100% |
| 2.0 | 100% | 100% |
| 2.2 | 100% | 100% |
| 2.4 | 99% | 100% |
| 2.8 | 100% | 99% |
| 3.2 | 96% | 100% |
| 3.6 | 85% | 84% |
| 4.0 | 76% | 74% |
