# Supplementary material for: High resolution time series reveals cohesive but short-lived communities in coastal plankton
Source: Nat Commun. 2018 Jan 18;9:266. doi: 10.1038/s41467-017-02571-4 (PMC5773528; doi:10.1038/s41467-017-02571-4)
Supplement: Supplementary file 3 — Description of Additional Supplementary Files [file 41467_2017_2571_MOESM3_ESM.pdf]

Description of Supplementary Files

File Name: Supplementary Data 1
Description: Counts of each bacterial OTU.

File Name: Supplementary Data 2
Description: Counts of each eukaryal OTU.

File Name: Supplementary Data 3
Description: Relative abundances of each bacterial OTU.

File Name: Supplementary Data 4
Description: Relative abundances of each eukaryal OTU.

File Name: Supplementary Data 5
Description: Representative sequences of each bacterial OTU.

File Name: Supplementary Data 6
Description: Representative sequences of each eukaryal OTU.

File Name: Supplementary Data 7
Description: Metadata values for each day.
